# Orthostatic Hypotension and the Long-Term Risk of Dementia: A Population-Based Study

**DOI:** 10.1371/journal.pmed.1002143

**Published:** 2016-10-11

**Authors:** Frank J. Wolters, Francesco U. S. Mattace-Raso, Peter J. Koudstaal, Albert Hofman, M. Arfan Ikram, Heart Brain Connection Collaborative Research Group

**Affiliations:** 1 Department of Epidemiology, Erasmus Medical Centre, Rotterdam, the Netherlands; 2 Department of Neurology, Erasmus Medical Centre, Rotterdam, the Netherlands; 3 Department of Geriatrics, Erasmus Medical Centre, Rotterdam, the Netherlands; 4 Department of Epidemiology, Harvard T.H. Chan School of Public Health, Boston, Massachusetts, United States of America; 5 Department of Radiology, Erasmus Medical Centre, Rotterdam, the Netherlands; University of Oxford, UNITED KINGDOM

## Abstract

**Background:**

Orthostatic hypotension (OH) is a common cause of transient cerebral hypoperfusion in the population. Cerebral hypoperfusion is widely implicated in cognitive impairment, but whether OH contributes to cognitive decline and dementia is uncertain. We aimed to determine the association between OH and the risk of developing dementia in the general population.

**Methods and Findings:**

Between 4 October 1989 and 17 June 1993, we assessed OH in non-demented, stroke-free participants of the population-based Rotterdam Study. OH was defined as a ≥20 mm Hg drop in systolic blood pressure (SBP) or ≥10 mm Hg drop in diastolic blood pressure (DBP) within 3 min from postural change. We furthermore calculated within participant variability in SBP related to postural change, expressed as coefficient of variation. Follow-up for dementia was conducted until 1 January 2014. We determined the risk of dementia in relation to OH and SBP variability, using a Cox regression model, adjusted for age; sex; smoking status; alcohol intake; SBP; DBP; cholesterol:high-density lipoprotein ratio; diabetes; body mass index; use of antihypertensive, lipid-lowering, or anticholinergic medication; and apolipoprotein E genotype. Finally, we explored whether associations varied according to compensatory increase in heart rate. Among 6,204 participants (mean ± standard deviation [SD] age 68.5 ± 8.6 y, 59.7% female) with a median follow-up of 15.3 y, 1,176 developed dementia, of whom 935 (79.5%) had Alzheimer disease and 95 (8.1%) had vascular dementia. OH was associated with an increased risk of dementia (adjusted hazard ratio [aHR] 1.15, 95% CI 1.00–1.34, *p =* 0.05), which was similar for Alzheimer disease and vascular dementia. Similarly, greater SBP variability with postural change was associated with an increased risk of dementia (aHR per SD increase 1.08, 95% CI 1.01–1.16, *p =* 0.02), which was similar when excluding those who fulfilled the formal criteria for OH (aHR 1.08, 95% CI 1.00–1.17, *p =* 0.06). The risk of dementia was particularly increased in those with OH who lacked a compensatory increase in heart rate (within lowest quartile of heart rate response: aHR 1.39, 95% CI 1.04–1.85, *p-*interaction = 0.05). Limitations of this study include potential residual confounding despite rigorous adjustments, and potentially limited generalisability to populations not of European descent.

**Conclusions:**

In this population predominantly of European descent, OH was associated with an increase in long-term risk of dementia.

## Abstract 2

Using data from the Rotterdam study, Mohammad Ikram and colleagues examine the association between orthostatic hypotension and the risk of developing dementia over 25 years.

## Author Summary

**Why Was This Study Done?:**

- Orthostatic hypotension is a common cause of transient cerebral hypoperfusion that is associated with subclinical brain disease, as well as increased risk of stroke.
- Hypoperfusion has been implicated in the pathophysiology of dementia, but whether orthostatic hypotension affects the risk of dementia is uncertain.

**What Did the Researchers Do and Find?:**

- Back in 1990, we measured orthostatic hypotension in 6,204 community-dwelling individuals participating in the Dutch population-based Rotterdam Study, and we followed these people for occurrence of dementia until 2014.
- Having orthostatic hypotension at start of the study increased the risk of developing dementia over the next 25 years by 15%, with similar results for all-cause dementia and Alzheimer disease.
- The risk of dementia was particularly increased when the orthostatic blood pressure drops were not compensated for by a sufficient increase in heart rate.

**What Do These Findings Mean?:**

- These findings suggest that transient cerebral hypoperfusion plays a role in the aetiology of dementia and that further studies are warranted to investigate the effects of hypoperfusion and treatment of orthostatic hypotension on markers of neurodegenerative disease and cognition.

## Introduction

Cardiovascular health is now well-established as a key determinant in the prevention of dementia, including Alzheimer disease [1,2], but the mechanisms by which vascular damage leads to cognitive decline remain largely unknown. As cerebral hypoperfusion is widely implicated in dementia [3,4], cerebral haemodynamics have been suggested as a potential link between vascular risk factors and dementia [5]. Two important mechanisms for maintenance of proper and continuous cerebral perfusion are local vasoreactivity and autonomous nervous system function. Cerebral vasoreactivity has indeed been found to be associated with the risk of developing dementia in the general population [6], but the role of autonomous nervous system function in the onset of dementia has been less well studied.

Autonomic dysfunction may result in orthostatic hypotension (OH), which affects 20%–30% of the elderly population [7,8]. OH is characterised by a marked drop in blood pressure following postural change, insufficiently compensated for by sympathetic and parasympathetic mechanisms. This drop in blood pressure may elicit transient cerebral hypoperfusion, especially in the absence of a compensatory increase in heart rate. OH is associated with an increased risk of cardiovascular events, stroke, and mortality [9]. Moreover, OH is highly prevalent among patients with dementia and mild cognitive impairment, compared to healthy controls [10–13], but only one study has assessed the longitudinal relation between OH and the risk of dementia in initially healthy participants. In this Swedish population, OH was associated with an increased risk of dementia at re-examination after 6 y, but the investigators were unable to adjust for cardiovascular risk factors aside from hypertension, and attrition was substantial, with 37.5% of participants lost to follow-up between examination rounds [14]. These limited data regarding OH and cognition prompted a recent review and meta-analysis to conclude that longitudinal studies using standardised criteria are needed to elucidate whether OH is an independent risk factor for developing dementia [9,15]. We therefore aimed to determine the association between OH and the risk of dementia, in a long-term, ongoing population-based study.

## Methods

### Ethics Statement

The Rotterdam Study has medical ethics committee approval per the Population Study Act Rotterdam Study, executed by the Ministry of Health, Welfare and Sports of the Netherlands. Written informed consent was obtained from all participants. For the current study, the analysis plan was drafted in June 2015.

### Study Population

This study is embedded within the Rotterdam Study, a large ongoing population-based cohort study in the Netherlands, with an initial study population of 7,983 participants (78% of invitees) aged ≥55 y from the Ommoord area, a suburb of Rotterdam. The Rotterdam Study methods have been described in detail previously [16]. In brief, participants were interviewed at home and subsequently examined at the research centre for baseline assessment from October 1989 to July 1993. OH was determined during baseline assessment. Of 7,983 interviewed participants, 7,157 (89.7%) visited the research centre for the baseline physical examination. As of 2015, five follow-up examinations have been carried out.

### Orthostatic Hypotension Assessment

Blood pressure and heart rate were measured using an automatic recorder (Dinamap, Critikon). The baseline blood pressure reading was the mean of two measurements on the right upper arm with the participant in supine position, after 5 min of rest. Measurements were repeated in the standing position after 1, 2, and 3 min. OH was defined as ≥20 mm Hg decrease in systolic blood pressure (SBP) or ≥10 mm Hg decrease in diastolic blood pressure (DBP) after postural change at any of the three measurements, in accordance with the Consensus Committee of the American Autonomic Society and the American Academy of Neurology [17,18]. We defined severity of OH by degree of blood pressure drop, i.e., ≥20/10 but <30/15, ≥30/15 but <40/20, and ≥40/20 mm Hg. We calculated continuous measures of blood pressure change in response to postural change, expressed as the coefficient of variation of within participant variability, defined as the ratio of the standard deviation (SD) to the mean of all measurements (i.e., measurements in supine and upright position combined). Furthermore, we determined the maximum increase in heart rate within 3 min after postural change. Directly afterwards, participants were asked whether they had felt unwell within the minutes following postural change.

### Dementia Screening and Cognitive Function Assessment

Participants were screened for dementia at baseline and follow-up examinations using a three-step protocol [19]. Screening was done using the Mini-Mental State Examination (MMSE) and the Geriatric Mental State Schedule (GMS) organic level. Those with MMSE < 26 or GMS > 0 subsequently underwent examination and informant interview using the Cambridge Examination for Mental Disorders of the Elderly (CAMDEX). Additionally, the total cohort was continuously monitored for dementia through computerised linkage of medical records from general practitioners and the regional institute for outpatient mental healthcare with the study database. Available neuroimaging data were used when required for establishing a diagnosis. For all suspected cases of dementia, a consensus panel led by a consultant neurologist (P. J. K.) decided on the final diagnosis in accordance with standard criteria for dementia (DSM-III-R), Alzheimer disease (NINCDS-ADRDA), and vascular dementia (NINDS-AIREN). Follow-up until 1 January 2014 was near complete (94.0% of potential person years), and participants were censored within this follow-up period at date of dementia diagnosis, date of death, date of loss to follow-up, or 1 January 2014, whichever came first.

### Other Measurements

We assessed smoking status (i.e., current, former, never), alcohol intake, use of antihypertensive medication, use of lipid-lowering medication, and use of anticholinergic medication at baseline by interview. Anticholinergic medication included antipsychotic and antidepressant medication, but also drugs prescribed against parkinsonism, urinary incontinence, or obstructive pulmonary disease that can have anticholinergic side effects. Fasting serum lipid levels were measured at baseline. Hypertension was defined as the use of antihypertensive medication and/or elevated systolic or DBP (>140/90 mm Hg). Body mass index (kg/m^2^) was computed from measurements of height and weight. Diabetes mellitus was defined as the use of blood-glucose-lowering medication at baseline or a random serum glucose level ≥11.1 mmol/l [20]. Myocardial infarction and atrial fibrillation were assessed by direct questioning and presence of abnormalities on a 12-lead electrocardiogram as determined by study physicians and reviewed by a cardiologist. Heart failure was determined using a validated score, similar to the definition of heart failure of the European Society of Cardiology [21]. *APOE* genotype was determined using polymerase chain reaction on coded DNA samples.

### Analysis

Analyses included all non-demented, stroke-free participants attending the study centre for examination. Of 7,157 participants attending the study centre, 531 were ineligible due to prevalent dementia (*n =* 312), stroke (*n =* 168), or both (*n =* 51). Missing covariate data (maximum 17.6%), except for *APOE* genotype, were imputed using 5-fold multiple imputation, based on determinant (presence of OH and postural SBP variability), outcome, and included covariates. Distribution of covariates was similar in the imputed versus non-imputed dataset.

We determined the association between presence of OH and incident dementia, using Cox proportional hazard models. We repeated the analysis with dementia or death as a joint outcome measure, to reduce selection due to competing risk (upon referee’s request). Subsequently, we analysed categories of increasing severity of orthostatic blood pressure drop, and OH with and without feeling unwell. Because of right-skewedness, we performed a natural logarithmic transformation of SBP variability to obtain a roughly normal distribution (mean −2.52, SD 0.58). *Z*-scores were computed by dividing the difference between the individual value and the population mean by the population SD. We determined the association between SBP variability related to postural change and incident dementia, per quartile and continuously per SD increase, using a Cox model. To eliminate a paradoxical impact of high blood pressure variability in those with excessive increases, we repeated analyses after excluding those with a ≥20 mm Hg increase in SBP or ≥10 mm Hg increase in DBP within 3 min (upon referee’s request). Furthermore, we determined whether associations extended to those without a formal diagnosis of OH. We then assessed whether the risk of dementia in relation to orthostatic blood pressure drops was modified by response in heart rate after postural change, by testing for multiplicative interaction in the above Cox model and providing risk estimates of OH for dementia per quartile of response in heart rate. We verified that the proportional hazard assumption was not violated in these models by plotting the partial (Schoenfeld) residuals against follow-up time.

All analyses were adjusted for age and sex (model I), and additionally in a second model for smoking status, alcohol intake, systolic and diastolic blood pressure, use of antihypertensive medication, ratio of serum total cholesterol to high-density lipoprotein, use of lipid-lowering medication, diabetes mellitus, body mass index, use of anticholinergic medication, and *APOE* genotype (model II).

We repeated the analyses for Alzheimer disease and vascular dementia separately, after censoring participants at time of incident stroke, after excluding those with Parkinson disease at baseline, after excluding those with heart disease (i.e., coronary heart disease, heart failure, atrial fibrillation; upon referee’s request), and after excluding those with possible postural tachycardia syndrome (defined as a ≥30 beats per minute increase in heart rate or any heart rate of ≥120 beats per minute). Finally, we performed several sensitivity analyses: (1) for men and women separately, (2) for persons above and below the median age (68.5 y), (3) excluding the first 5 y of follow-up to assess for reverse causality, (4) for those with and without heart failure at baseline, (5) for those with and without a history of hypertension, (6) distinguishing use of antihypertensive drugs, and (7) for those with and without diabetes (upon referee’s request). Finally, we repeated analyses for OH, SBP variability, and dementia risk in a subset of participants without significant comorbidity (i.e., excluding those with heart disease, Parkinson disease, and diabetes; upon referee’s request).

All analyses were done using IBM SPSS Statistics version 23.0. Alpha level (type 1 error) was set at 0.05.

## Results

Of 6,626 eligible participants, 6,303 (95.1%) underwent examination for OH. No baseline blood pressure measurement was obtained in eight individuals, and no measurement after postural change in 91 individuals, leaving a total of 6,204 (93.6%) cases for analysis. Baseline characteristics of participants are shown in Table 1.

**Table 1:** Baseline characteristics (*n* = 6,204).

Characteristic	Value
**Age**	68.5 ± 8.6
**Female sex**	3,704 (59.7%)
**Systolic blood pressure (mm Hg)**	139 ± 22
**Diastolic blood pressure (mm Hg)**	74 ± 11
**Antihypertensive medication**	1,901 (30.7%)
**Diabetes mellitus**	421 (7.2%)
**Body mass index (kg/m** ^**2**^ **)**	26.3 ± 3.6
**Serum cholesterol (mmol/l)**	6.6 ± 1.2
**Serum high-density lipoprotein (mmol/l)**	1.4 ± 0.4
**Lipid-lowering medication**	150 (2.4%)
**Smoking**	
Former	2,495 (41.9%)
Current	1,257 (21.1%)
**Alcohol intake (g/d), median (IQR)**	3.4 (0.2–14.8)
**Anticholinergic medication**	1,391 (22.4%)
***APOE* genotype**	
ε3/ε3	3,457 (58.3%)
ε2/ε2, ε2/ε3, or ε2/ε4	978 (16.4%)
ε3/ε4 or ε4/ε4	1,494 (25.3%)
**Orthostatic hypertension**	1,152 (18.6%)
≥20/10 mm Hg, but <30/15 mm Hg	773 (12.5%)
≥30/15 mm Hg, but <40/20 mm Hg	239 (3.9%)
≥40/20 mm Hg	140 (2.3%)
**Blood pressure variability** *, **median (IQR)**	0.08 (0.06–0.12)

Non-imputed data presented as frequency (percent) for categorical values and mean ± SD for continuous variables, unless indicated otherwise.

*Expressed as coefficient of variation.

IQR, interquartile range.

**Fig 1:**
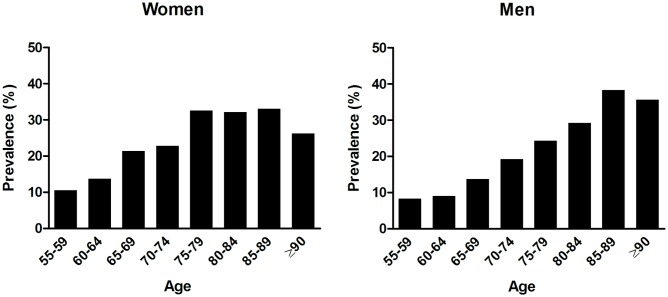
Age-specific prevalence of orthostatic hypotension in men and women.

Overall, 1,152/6,204 (18.6%) participants had OH. The prevalence of OH steeply increased with age, to 30.6% of those aged ≥75 y. Although prevalence in the elderly was similar for men and women, there was a slightly higher prevalence in women at younger ages (Fig 1). Of all patients with OH, 160 (13.9%) reported feeling unwell along with their blood pressure drop.

During a median follow-up time of 15.3 y (interquartile range 8.3–20.8), 1,176 individuals developed dementia, of whom 935 (79.5%) were diagnosed with Alzheimer disease, 95 (8.1%) with vascular dementia, 43 (3.7%) with Parkinson dementia, and 30 (2.6%) with another type of dementia, and in 73 (6.2%) no definite subdiagnosis could be made. Of all incident dementia cases, 129 were preceded by a stroke a median 3.7 y (interquartile range 1.2–7.2) before diagnosis of dementia.

**Fig 2:**
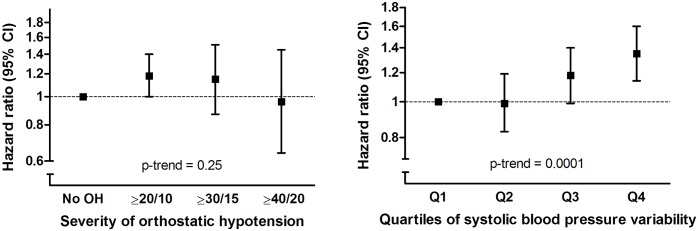
Risk of dementia in relation to severity of orthostatic blood pressure drop (in mm Hg) and quartiles of systolic blood pressure variability. OH, orthostatic hypotension.

OH at baseline was associated with an increased risk of dementia during follow-up (adjusted hazard ratio [aHR] 1.15, 95% CI 1.00–1.34, *p =* 0.05; Table 2). Similarly on a continuous scale, variability in SBP related to postural change was associated with an increased risk of dementia (aHR per SD increase: 1.08, 95% CI 1.01–1.16, *p =* 0.02). This association was similar when excluding those who fulfilled the formal criteria for OH (aHR 1.08, 95% CI 1.00–1.17) and unaltered by excluding those with a marked increase in blood pressure following postural change (see S1 Table). Results were similar for Alzheimer disease only. For vascular dementia, we observed higher risk estimates with OH than for Alzheimer disease in the age- and sex-adjusted model (aHR 1.53, 95% CI 0.97–2.43), but these were largely explained by cardiovascular risk factors, so that fully adjusted estimates were similar to those for Alzheimer disease (aHR 1.20, 95% CI 0.73–1.96; Table 2). We did not observe a clear exposure-response relation for severity of OH, because of lower effect estimates for participants with the most severe blood pressure drops (Fig 2). In contrast, the risk of dementia strongly increased per quartile of blood pressure variability (Fig 2; for a full table with risk estimates per quartile and per SD, see S2 Table). Risk estimates were similar when modelling dementia or death as a joint outcome (OH: aHR 1.17, 95% CI 1.08–1.27; blood pressure variability: aHR 1.08, 95% CI 1.04–1.12). Estimates for both OH and SBP variability were attenuated when incorporating these simultaneously in a model (OH: aHR 1.07, 95% CI 0.90–1.27; blood pressure variability: aHR 1.06, 95% CI 0.99–1.14).

**Table 2:** Orthostatic hypotension and the risk of dementia.

Model and Outcome	All Dementia (*n/N* = 1,176/6,204)		Alzheimer Disease (*n/N* = 935/6,204)		Vascular Dementia (*n/N* = 95/6,204)	
	aHR, 95% CI	*p*-Value	aHR, 95% CI	*p*-Value	aHR, 95% CI	*p*-Value
**Model I**						
OH (yes versus no)	1.14, 0.99–1.31	0.08	1.11, 0.95–1.30	0.11	1.53, 0.97–2.43	0.07
SBP variability (per SD*)	1.07, 1.00–1.14	0.04	1.10, 1.03–1.18	0.008	0.93, 0.76–1.13	0.47
**Model II**						
OH (yes versus no)	1.15, 1.00–1.34	0.05	1.17, 0.99–1.37	0.07	1.20, 0.73–1.96	0.48
SBP variability (per SD*)	1.08, 1.01–1.16	0.02	1.11, 1.04–1.20	0.003	0.92, 0.76–1.13	0.43

Model I: adjusted for age and sex. Model II: model I with additional adjustment for systolic and diastolic blood pressure, antihypertensive medication, diabetes, ratio of serum total cholesterol to high-density lipoprotein, lipid-lowering medication, smoking status, alcohol intake, anticholinergic medication, body mass index, and *APOE* genotype.

*Per SD increase in coefficient of variation.

aHR, adjusted hazard ratio; OH, orthostatic hypotension; SBP, systolic blood pressure; SD, standard deviation.

**Fig 3:**
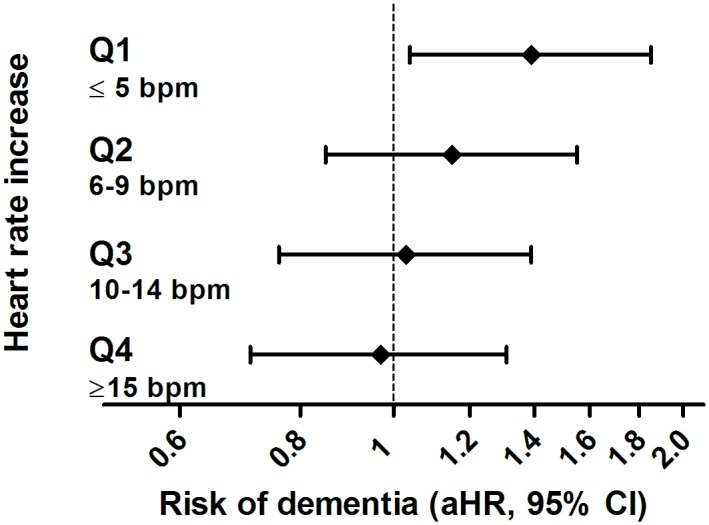
Risk of dementia in relation to orthostatic hypotension, stratified per quartile of response in heart rate. aHR, adjusted hazard ratio; bpm, beats per minute.

Results for OH where individuals reported feeling unwell along with the blood pressure drop were similar to results for OH without feeling unwell (aHR 1.20, 95% CI 0.86–1.66, versus aHR 1.15, 95% CI 0.98–1.34, respectively). The risk of dementia related to OH was most profound in participants who lacked a compensatory increase in heart rate (aHR for lowest quartile of heart rate response 1.39, 95% CI 1.04–1.85, *p-*value for interaction = 0.05; Fig 3). This risk was similar after excluding all participants taking beta-blockers (see S2 Table).

Sensitivity analyses showed similar results after censoring for incident stroke, excluding participants with prevalent Parkinson disease, excluding those with possible postural tachycardia syndrome, or omitting the first 5 y of follow-up (Table 3). A history of hypertension or use of any antihypertensive medication did not modify the risk of dementia associated with OH (Table 3). Amongst 177 participants with heart failure at baseline, risk estimates for OH were higher than in those without heart failure, albeit not statistically significantly (aHR 1.52, 95% CI 0.63–3.66, *p-*value for interaction = 0.07). Risk estimates for both OH and SBP variability were slightly stronger when excluding participants with cardiac disease, neurodegenerative comorbidity, or diabetes all together (see S3 Table).

**Table 3:** Sensitivity analyses for the association between orthostatic hypotension and incident dementia.

Sensitivity/Subgroup Analysis	*n/N*	Adjusted Hazard Ratio, 95% CI
**Censoring for incident stroke**	1,001/5,929	1.18, 1.01–1.38
**Excluding history of Parkinson disease**	1,076/5,704	1.16, 1.00–1.35
**Excluding history of heart disease** *	946/5,018	1.28, 1.09–1.50
**Excluding possible postural tachycardia syndrome** **	1,104/5,775	1.18, 1.02–1.37
**Excluding the first 5 y of follow-up**	882/5,081	1.22, 1.03–1.44
**Sex**		
Male	344/2,415	1.04, 0.77–1.41
Female	784/3,514	1.19, 1.00–1.40
**Age (stratified by median)**		
<68.5 y	388/3,186	1.05, 0.78–1.41
≥68.5 y	740/2,742	1.16, 0.98–1.38
**Heart failure**		
No	1,089/5,685	1.13, 0.97–1.31
Yes	30/177	1.52, 0.63–3.66
**Hypertension**		
No	469/2,674	1.22, 0.96–1.55
Yes	657/3,244	1.12, 0.93–1.36
**Antihypertensive medication**		
None	768/4,104	1.13, 0.94–1.36
Any antihypertensive drug	360/1,825	1.15, 0.90–1.47
**Diabetes**		
No	958/5,018	1.12, 0.94–1.34
Yes	170/911	1.35, 0.74–2.47

*Includes myocardial infarction, heart failure, and atrial fibrillation.

**Defined as a ≥30 beats per minute increase in heart rate or any heart rate ≥120 beats per minute.

## Discussion

In this large population-based study, OH was present in nearly one in five participants and was associated with a 15% increase in long-term risk of dementia. The risk of developing dementia was highest in those with OH lacking a compensatory increase in heart rate. Similarly, higher variability in blood pressure related to postural change was associated with a higher risk of dementia, even in individuals without a formal diagnosis of OH.

Prevalence of OH in our study was high and increased steeply with age, in line with previous studies among individuals within a similar age range in the community [7,8]. A few studies have investigated OH in relation to cognitive test performance. In the ARIC study, OH was associated with decline on two cognitive tests, but this decline was largely explained by cardiovascular risk factors [22]. Two smaller studies found no overall association between OH and decline on the MMSE after 2 y [7,23]. Conversely, OH was found to increase the risk of conversion from mild cognitive impairment to dementia after 3 y [24], as well as the risk of dementia in patients with Parkinson disease [25]. Only one other study has assessed the relation between OH and the risk of dementia in initially healthy individuals. In a sample of 1,480 individuals in the Swedish general population, OH was associated with the risk of having dementia at re-examination after 6 y [14]. However, the investigators were unable to fit survival models or adjust for cardiovascular risk factors aside from hypertension, and attrition was substantial, with 37.5% of participants lost to follow-up [14]. We found OH to be associated with long-term risk of dementia on continuous follow-up, independent of various other risk factors.

The most apparent explanation for our findings is that OH causes brain damage due to recurrent transient cerebral hypoperfusion. Autonomic nervous system function is responsible for maintaining continuous cerebral perfusion, together with local vasoreactivity, which has previously been associated with the risk of dementia in the general population [6]. Brief episodes of hypoperfusion elicited by sudden blood pressure drops may lead to hypoxia, with detrimental effects on brain tissue via, for instance, neuroinflammation and oxidative stress [26]. These mechanisms have been suggested to be of particular relevance in the pathogenesis of small vessel disease [27], and orthostatic blood pressure drops in patients with dementia have been associated with deep white matter and basal ganglia hyperintensities [28], albeit not with overall white matter lesion volume [29]. The reduction in cerebral blood flow with autonomic failure has also been reported to predominantly affect the hippocampus [30], possibly linking hypoperfusion to early Alzheimer pathology. Another potential explanation for our findings might be that OH serves as a marker of other detrimental consequences of autonomic dysfunction, such as blood pressure variability [31,32], response to Valsalva manoeuvre [13,33], cardiovascular reflex and heart rate variability [34,35], and 30/15 ratio [35]. Several of these measures may be linked to dementia via hypoperfusion, but other pathways could be involved also. For instance, decreased arterial wall compliance with hypertension likely contributes to OH by diminishing baroreceptor sensitivity [36]. Arterial stiffness and OH are both associated with increased burden of cerebral white matter lesions and vascular disease including stroke [9,28,37,38]. As these are established risk factors for dementia, these conditions may act as mediators or indicate shared aetiology between diseases. Nevertheless, we did not see risk estimates for all-cause dementia attenuated after adjustment for cardiovascular risk factors, censoring for clinical stroke, or restricting the population to those without cardiac or neurodegenerative comorbidities. Alternatively, sympathetic failure can occur with diabetic neuropathy, and although we adjusted for clinical history of diabetes, some patients with impaired fasting glucose might already have had autonomic derailment together with subclinical small vessel disease. We had no other direct measures of autonomic dysfunction to ascertain whether OH is the main driving force behind our findings. Blood pressure variability in our study was measured following postural change and therefore reflects a distinct orthostatic response, although it might in part occur as a manifestation of wider autonomic failure, unrelated to postural change. Accordingly, risk estimates for both OH and blood pressure variability attenuated after incorporating both in the same model, but it remains unclear whether this is because they are both computed from postural-change-related measurements or because they reflect the same underlying autonomic dysfunction. Given the interaction we found between OH and the lack of a compensatory increase in heart rate, the impact of OH may well vary with the degree of overall autonomic failure, and future studies are encouraged to incorporate various measures of autonomic dysfunction collected in the same individuals simultaneously (for which reported risks and effect estimates may serve as a guidance). Although autonomic dysfunction may even reflect early signs of neurodegeneration, the long follow-up duration of our study renders reverse causality less likely.

The risk of dementia associated with OH in our study was independent of whether participants reported feeling unwell along with the blood pressure drop, and the vast majority of patients with OH did not have symptoms during testing. Although for blood pressure variability we observed an exposure-response association, we did not find this for severity of OH itself. As OH is also associated with mortality [9], this finding may be attributable to competing risk, causing the most severely affected participants to die at a younger age, prior to developing dementia. Alternatively, rather than the degree of blood pressure drop, the lack of compensatory increase in heart rate may better reflect the severity of consequences of orthostatic drops in blood pressure. Taken together, our findings suggest that formal assessment of OH is necessary to have sufficient test sensitivity, and incorporation of heart rate response in the definition of OH may contribute to evaluating aetiology and clinical severity. Hypotension might be harmful even without accompanying clinical symptoms such as light-headedness. This lack of symptoms with orthostasis was previously observed in patients with dementia [39] and may warrant caution in view of studies linking low blood pressure in late life to cognitive decline and dementia [40].

OH most commonly arises due to autonomic dysfunction in the absence of neurological disease, but may be provoked by synucleinopathies (e.g., Parkinson disease), small fibre peripheral neuropathy, volume depletion (e.g., due to diuretics), and diminished cardiac pump function. In addition, several drugs can cause or aggravate OH, including antihypertensive agents and antidepressants. Participants in our study with heart failure at baseline seemed particularly affected by OH, possibly due to the lack of a compensatory increase in stroke volume. OH has been associated with the development of structural cardiac changes, including left ventricular hypertrophy [41], which may function as a mediator towards dementia [42,43]. However, the subgroup of participants with heart failure in our study was too small to draw any firm conclusions. We found similar associations between OH and dementia after excluding those with Parkinson disease, and in users versus non-users of antihypertensive medication.

Although we believe our findings are valid, there are certain limitations to our study to take into account. First, measures of OH were not available for all eligible participants. Although this was largely due to logistic reasons, we cannot completely rule out selection bias. Second, despite adjustment for many potentially confounding factors, residual confounding may still occur. However, given the lack of attenuation of our results in our second, more fully adjusted model, residual confounding is unlikely to result from the most relevant, included covariates. Third, we continued blood pressure measurements for up to 3 min after postural change, and while this approach is in line with international guidelines, it may have resulted in missed orthostatic blood pressure drops beyond this time window [44]. However, any misclassification (i.e., missed diagnosis of OH) would likely have led to underestimation of the true effect. Fourth, subtypes of dementia were based on clinical diagnosis, and mixed pathology (e.g., Lewy bodies) in patients with clinical Alzheimer disease may contribute to the observed associations. Fifth, we were unable to adjust for the fact that OH predisposes for falls, which may contribute to cognitive decline due to traumatic brain injury. Finally, the majority of our study population was of European descent, and findings may not be applicable to other ethnicities.

In conclusion, OH is associated with an increased risk of dementia in the general population. This finding supports an important role for maintaining continuous cerebral perfusion in the prevention of dementia.

## Supporting Information

S1 STROBE checklist (DOC)

S1 Table Systolic blood pressure variability in relation to the risk of dementia in subgroups of participants who did not meet the formal criteria for orthostatic hypotension, and excluding those with a strong increase in blood pressure following postural change. (DOCX)

S2 Table Orthostatic hypotension and the risk of dementia by heart rate increase, in the total study population and after excluding participants who used beta-blockers. (DOCX)

S3 Table Orthostatic hypotension, systolic blood pressure variability, and the risk of dementia in the subset of participants without myocardial infarction, heart failure, atrial fibrillation, Parkinson disease, or diabetes. (DOCX)

## Abbreviations

aHR: adjusted hazard ratio
DSP: diastolic blood pressure
OH: orthostatic hypotension
MMSE: Mini-Mental State Examination
SD: standard deviation
SBP: systolic blood pressure
