# Supplementary material for: Orthostatic Hypotension and the Long-Term Risk of Dementia: A Population-Based Study
Source: PLoS Med. 2016 Oct 11;13(10):e1002143. doi: 10.1371/journal.pmed.1002143 (PMC5058559; doi:10.1371/journal.pmed.1002143)
Supplement: S1 STROBE checklist — (DOC) [file pmed.1002143.s001.doc]

STROBE Statement—checklist of items that should be included in reports of observational studies

|  | Item No | Recommendation |
| --- | --- | --- |
| **Title and abstract** | 1✓abstract+title  abstract+title | (*a*) Indicate the study’s design with a commonly used term in the title or the abstract |
|  |  | (*b*) Provide in the abstract an informative and balanced summary of what was done and what was found |
| Introduction | | |
| Background/rationale | 2✓ whole introduction | Explain the scientific background and rationale for the investigation being reported |
| Objectives | 3✓paragraph 2 | State specific objectives, including any prespecified hypotheses  “We therefore aimed to determine the association between orthostatic hypotension and the risk of dementia, in a long-term, ongoing population-based study.” |
| Methods | | |
| Study design | 4✓ paragraph 2 | Present key elements of study design early in the paper  Second paragraph of methods section: “This study is embedded within the Rotterdam Study, a large ongoing population-based cohort study in the Netherlands, with an initial study population of 7983 participants (78% of invitees) aged ≥55 years from the Ommoord area, a suburb of Rotterdam. The Rotterdam Study methods have been described in detail previously.[16] In brief, participants were interviewed at home and subsequently examined at the research centre for baseline assessment from March 1990 to July 1993.” |
| Setting | 5✓ paragraph 2+4 | Describe the setting, locations, and relevant dates, including periods of recruitment, exposure, follow-up, and data collection  “Participants were screened for dementia at baseline and follow-up examinations using a three-step protocol […] Follow-up was near complete until 1st January 2014 (94.0% of potential person years)” |
| Participants | 6✓ paragraph 2+6 and results paragraph 1 | (*a*) *Cohort study*—Give the eligibility criteria, and the sources and methods of selection of participants. Describe methods of follow-up  See above + “Analyses included all non-demented, stroke-free participants attending the study centre for examination. Of 7157 participants attending the study centre, 531 were ineligible due to prevalent dementia (n=312), stroke (n=168), or both (n=51).”  *Case-control study*—Give the eligibility criteria, and the sources and methods of case ascertainment and control selection. Give the rationale for the choice of cases and controls  *Cross-sectional study*—Give the eligibility criteria, and the sources and methods of selection of participants |
|  |  | (*b*) *Cohort study*—For matched studies, give matching criteria and number of exposed and unexposed. Please see tables +  “Overall, 1152/6204 (18.6%) participants had orthostatic hypotension. The prevalence of orthostatic hypotension steeply increased with age, to 30.6% of those aged >75 years”  *Case-control study*—For matched studies, give matching criteria and the number of controls per case |
| Variables | 7✓ paragraph 5 | Clearly define all outcomes, exposures, predictors, potential confounders, and effect modifiers. Give diagnostic criteria, if applicable  “we assessed whether the risk of dementia in relation to orthostatic blood pressure drops was modified by response in heart rate after postural change, by testing for multiplicative interaction”  “All analyses were adjusted for age and sex, and additionally in a second model for smoking habits, alcohol intake, systolic and diastolic blood pressure, use of antihypertensive medication, serum total cholesterol and HDL, use of lipid-lowering medication, diabetes mellitus, body mass index, neuroleptic and neuroanaleptic medication, and APOE genotype. To rule out the effect of stroke, for this second model we censored participants at time of incident stroke during follow-up.” |
| Data sources/ measurement | 8✓throughout methods section | For each variable of interest, give sources of data and details of methods of assessment (measurement). Describe comparability of assessment methods if there is more than one group |
| Bias | 9✓ paragraph 2+5+6 and results paragraph 1 | Describe any efforts to address potential sources of bias  Please see above for mention of all confounders, and detailed information about cohort selection to provide information about potential selection. We provide a description of standardised measurement of orthostatic hypotension also. |
| Study size | 10✓ paragraph 2+5+6 and results paragraph 1 | Explain how the study size was arrived at |
| Quantitative variables | 11✓ throughout methods section | Explain how quantitative variables were handled in the analyses. If applicable, describe which groupings were chosen and why. Heart rate response categorised by quartiles (most objective); analyses for SBP variability per standard deviation as well as per quartile. |
| Statistical methods | 12✓ paragraph 6 | (*a*) Describe all statistical methods, including those used to control for confounding |
|  |  | (*b*) Describe any methods used to examine subgroups and interactions  See above for heart rate |
|  |  | (*c*) Explain how missing data were addressed  “Missing covariate data (maximum 17.6%) were imputed using 5-fold multiple imputation, based on determinant, outcome and included covariates. Distribution of covariates was similar in the imputed versus non-imputed dataset.” |
|  |  | (*d*) *Cohort study*—If applicable, explain how loss to follow-up was addressed  “Follow-up was near complete until 1st January 2014 (94.0% of potential person years), and participants were censored within this follow-up period at date of dementia diagnosis, date of death, date of loss to follow-up, or 1st January 2014, whichever came first.”  *Case-control study*—If applicable, explain how matching of cases and controls was addressed  *Cross-sectional study*—If applicable, describe analytical methods taking account of sampling strategy |
|  |  | (*e*) Describe any sensitivity analyses  “Finally, we performed several sensitivity analyses: 1) for men and women separately, 2) for persons above and below the median age (68.5 years), 3) excluding the first 5 years of follow-up to assess for reverse causality, 4) for those with and without heart failure at baseline, 5) for those with and without a history of hypertension, and 6) distinguishing use of (various classes of) anti-hypertensive drugs.” |

Continued on next page

| Results | | |
| --- | --- | --- |
| Participants | 13✓paragraph 1 | (a) Report numbers of individuals at each stage of study—eg numbers potentially eligible, examined for eligibility, confirmed eligible, included in the study, completing follow-up, and analysed  “Of 6626 eligible participants, 6303 (95.1%) underwent examination for orthostatic hypotension. No baseline blood pressure measurement was obtained in 8 individuals, and no measurement after postural change in 91 individuals, leaving a total of 6204 (93.6%) cases for analysis.” |
|  |  | (b) Give reasons for non-participation at each stage |
|  |  | (c) Consider use of a flow diagram |
| Descriptive data | 14✓ Table 1 | (a) Give characteristics of study participants (eg demographic, clinical, social) and information on exposures and potential confounders. Table 1 |
|  |  | (b) Indicate number of participants with missing data for each variable of interest |
|  |  | (c) *Cohort study*—Summarise follow-up time (eg, average and total amount) |
| Outcome data | 15✓paragraph 2+3 | *Cohort study*—Report numbers of outcome events or summary measures over time  “During a median follow-up time of 15.3 (IQR 8.3-20.8) years, 1176 individuals developed dementia, of whom 935 (79.5%) were diagnosed with Alzheimer’s disease, 95 (8.1%) vascular dementia, 43 (3.7%) Parkinson’s dementia, 30 (2.6%) another type of dementia, and in 73 (6.2%) no definite subdiagnosis could be made.” |
|  |  | *Case-control study—*Report numbers in each exposure category, or summary measures of exposure |
|  |  | *Cross-sectional study—*Report numbers of outcome events or summary measures |
| Main results | 16✓ Table 2 | (*a*) Give unadjusted estimates and, if applicable, confounder-adjusted estimates and their precision (eg, 95% confidence interval). Make clear which confounders were adjusted for and why they were included. Table 2 |
|  |  | (*b*) Report category boundaries when continuous variables were categorized |
|  |  | (*c*) If relevant, consider translating estimates of relative risk into absolute risk for a meaningful time period |
| Other analyses | 17✓ paragraph 4-6 and Table 3 | Report other analyses done—eg analyses of subgroups and interactions, and sensitivity analyses. See above. |
| Discussion | | |
| Key results | 18✓ paragraph 1+7 | Summarise key results with reference to study objectives  “In this large population-based study, orthostatic hypotension was present in nearly 1 in 5 participants, and associated with a 17% increase in long-term risk of dementia. The risk of developing dementia was highest in those with orthostatic hypotension lacking compensatory increase in heart rate. Similarly, higher variability in blood pressure related to postural change, was associated with a higher risk of dementia, even in those persons without a formal diagnosis of orthostatic hypotension.” |
| Limitations | 19✓paragraph 6 | Discuss limitations of the study, taking into account sources of potential bias or imprecision. Discuss both direction and magnitude of any potential bias  “First, measures of orthostatic hypotension were not available for all participants. Although this was largely due to logistic reasons, we cannot completely rule out selection bias. Second, we continued blood pressure measurements for up to three minutes after postural change, and while in line with international guidelines, this may have resulted in missed orthostatic blood pressure drops beyond this time window.(43) However, any misclassification (i.e. missed diagnosis of orthostatic hypotension) would likely have led to underestimation of the true effect. Third, subtypes of dementia were based on clinical diagnosis, and mixed pathology (e.g. Lewy bodies) in patients with clinical Alzheimer’s disease may contribute to the observed associations. Fourth, we were unable to adjust for the fact that orthostatic hypotension predisposes for falls, which may contribute to cognitive decline due to traumatic brain injury. Finally, the majority of our study population was of Caucasian descent, and findings may not be applicable to other ethnicities.” |
| Interpretation | 20✓ paragraph 7 | Give a cautious overall interpretation of results considering objectives, limitations, multiplicity of analyses, results from similar studies, and other relevant evidence |
| Generalisability | 21✓ paragraph 6 | Discuss the generalisability (external validity) of the study results  Sample from general population renders excellent generalisability |
| Other information | | |
| Funding | 22✓ page 20 | Give the source of funding and the role of the funders for the present study and, if applicable, for the original study on which the present article is based |

*Give information separately for cases and controls in case-control studies and, if applicable, for exposed and unexposed groups in cohort and cross-sectional studies.

**Note:** An Explanation and Elaboration article discusses each checklist item and gives methodological background and published examples of transparent reporting. The STROBE checklist is best used in conjunction with this article (freely available on the Web sites of PLoS Medicine at http://www.plosmedicine.org/, Annals of Internal Medicine at http://www.annals.org/, and Epidemiology at http://www.epidem.com/). Information on the STROBE Initiative is available at [www.strobe-statement.org](http://www.strobe-statement.org/)

The Rotterdam Study was designed in 1989 to investigate the aetiology (in mid-life) and disease course of chronic conditions in the elderly. Two of its primary focusses were stroke and dementia. A measurement of orthostatic hypotension, as described was implemented directly, as this was seen as a potentially interesting determinant for the risk of dementia. Now that we had long-term follow-up available we made more detailed plans for analyses. In addition to a dichotomised measure of orthostatic hypotension, we wanted to investigate the impact of blood pressure changes related to postural change across the full spectrum of exposure and hence computed within-subject variability of BP measurements. This also allowed us to explore the association of the exposure in people who did not fulfil the formal criteria for orthostatic hypotension. Furthermore, given the hypothesis that hypoperfusion explains the association between orthostatic hypotension and increase in heart rate can compensate for transient drops in blood pressure, we assessed heart rate as a potential effect modifier. Potential confounders and subgroups for sensitivity analyses were extracted from the literature, and selected on the basis of potential physiological mechanisms.
