# Supplementary material for: Orthostatic Hypotension and the Long-Term Risk of Dementia: A Population-Based Study
Source: PLoS Med. 2016 Oct 11;13(10):e1002143. doi: 10.1371/journal.pmed.1002143 (PMC5058559; doi:10.1371/journal.pmed.1002143)
Supplement: S1 Table — (DOCX) [file pmed.1002143.s002.docx]

**Supplemental Table 1.** Systolic blood pressure variability in relation to the risk of dementia.

|  | **All participants** | | | **Without orthostatic hypotension** | | | **Excluding strong increase in blood pressure*** | | |
| --- | --- | --- | --- | --- | --- | --- | --- | --- | --- |
|  | n/N | HR, 95% CI | P-value | n/N | HR, 95% CI | P-value | n/N | HR, 95% CI | P-value |
| SBP variability (CV) |  |  |  |  |  |  |  |  |  |
| Lowest quartile | 234/1389 | reference |  | 223/1325 | reference | - | 214/1251 | reference | - |
| 2^nd^ quartile | 224/1375 | 0.99, 0.83-1.19 | 0.92 | 210/1315 | 0.97, 0.80-1.17 | 0.71 | 210/1254 | 1.00, 0.82-1.21 | 0.97 |
| 3^rd^ quartile | 269/1375 | 1.18, 0.99-1.40 | 0.07 | 236/1210 | 1.18, 0.98-1.42 | 0.08 | 240/1221 | 1.14, 0.95-1.38 | 0.15 |
| 4^th^ quartile | 313/1367 | 1.35, 1.14-1.60 | 0.001 | 142/640 | 1.46, 1.18-1.81 | 0.001 | 278/1224 | 1.31, 1.09-1.57 | 0.003 |
| P-trend |  |  | 0.0001 |  |  | 0.0003 |  |  | 0.001 |
| Per SD | 1040/5506 | 1.08, 1.01-1.15 | 0.02 | 811/4490 | 1.08, 1.00-1.17 | 0.06 | 942/4950 | 1.08, 1.01-1.15 | 0.03 |

SBP=systolic blood pressure; CV=coefficient of variation; SD=standard deviation; HR=hazard ratio; CI=confidence interval.

* Defined as ≥20mmHg systolic, or ≥10mmHg diastolic increase

Model adjusted for age, sex, systolic and diastolic blood pressure, antihypertensive medication, diabetes, serum cholesterol and HDL, lipid-lowering medication, smoking, alcohol consumption, anti-cholinergic medication, and *APOE* genotype
