# Supplementary material for: Orthostatic Hypotension and the Long-Term Risk of Dementia: A Population-Based Study
Source: PLoS Med. 2016 Oct 11;13(10):e1002143. doi: 10.1371/journal.pmed.1002143 (PMC5058559; doi:10.1371/journal.pmed.1002143)
Supplement: S2 Table — (DOCX) [file pmed.1002143.s003.docx]

**Supplemental Table 2.** Orthostatic hypotension and the risk of dementia by heart rate increase.

|  | n/N | All participants  Adjusted HR, 95% CI for OH vs. no OH | P-value | Excluding beta-blocker users  Adjusted HR, 95% CI for OH vs. no OH | P-value |
| --- | --- | --- | --- | --- | --- |
| Heart rate increase |  |  |  |  |  |
| Lowest quartile (≤5 bpm) | 270/1378 | 1.39, 1.04-1.85 | 0.03 | 1.36, 0.95-1.94 | 0.09 |
| 2^nd^ quartile (6-9 bpm) | 297/1443 | 1.15, 0.85-1.55 | 0.38 | 1.25, 0.89-1.74 | 0.20 |
| 3^rd^ quartile (10-14 bpm) | 298/1634 | 1.03 0.76-1.39 | 0.84 | 1.06, 0.77-1.44 | 0.74 |
| 4^th^ quartile (≥15 bpm) | 262/1462 | 0.97, 0.71-1.31 | 0.83 | 0.99, 0.72-1.36 | 0.95 |

bpm=beats per minute; HR=hazard ratio; CI=confidence interval.

Model adjusted for age, sex, systolic and diastolic blood pressure, antihypertensive medication, diabetes, serum cholesterol and HDL, lipid-lowering medication, smoking, alcohol consumption, anti-cholinergic medication, and *APOE* genotype
