# Supplementary material for: Orthostatic Hypotension and the Long-Term Risk of Dementia: A Population-Based Study
Source: PLoS Med. 2016 Oct 11;13(10):e1002143. doi: 10.1371/journal.pmed.1002143 (PMC5058559; doi:10.1371/journal.pmed.1002143)
Supplement: S3 Table — (DOCX) [file pmed.1002143.s004.docx]

**Supplemental Table 3.** Orthostatic hypotension, systolic blood pressure variability, and the risk of dementia in the subset of participants without myocardial infarction, heart failure, atrial fibrillation, Parkinson’s disease, or diabetes.

|  | **All dementia (n/N=805/4372)** | **Alzheimer’s disease (n/N=648/4372)** | **Vascular dementia (n/N=64/4372)** |
| --- | --- | --- | --- |
|  | HR, 95% CI; p-value | HR, 95% CI; p-value | HR, 95% CI; p-value |
| **Model I** |  |  |  |
| Orthostatic hypotension (yes versus no) | 1.22, 1.02-1.46  0.03 | 1.19, 0.97-1.45  0.11 | 1.87, 1.05-3.34  0.04 |
| Systolic blood pressure variability (per SD*) | 1.07, 0.99-1.15 | 1.10, 1.01-1.19 | 0.95, 0.74-1.22 |
|  | 0.08 | 0.04 | 0.67 |
| **Model II** |  |  |  |
| Orthostatic hypotension (yes versus no) | 1.24, 1.03-1.50  0.02 | 1.23, 1.00-1.52  0.05 | 1.60, 0.86-2.97  0.14 |
| Systolic blood pressure variability (per SD*) | 1.07, 0.99-1.15  0.09 | 1.10, 1.00-1.20  0.04 | 0.95, 0.73-1.22  0.67 |

Model I adjusted for age and sex

Model II adjusted for age, sex, systolic and diastolic blood pressure, antihypertensive medication, diabetes, serum cholesterol and HDL, lipid-lowering medication, smoking, alcohol consumption, anti-cholinergic medication, and *APOE* genotype
